# Constitutive Androstane Receptor Agonist, TCPOBOP: Maternal Exposure Impairs the Growth and Development of Female Offspring in Mice

**DOI:** 10.3390/ijms24032602

**Published:** 2023-01-30

**Authors:** Shijia Pan, Yuan Guo, Wen Yu, Jia Zhang, Xiaoxiao Qiao, Letong Li, Pengfei Xu, Yonggong Zhai

**Affiliations:** 1 Beijing Key Laboratory of Gene Resource and Molecular Development, College of Life Sciences, Beijing Normal University, Beijing 100875, China; 2 Key Laboratory for Cell Proliferation and Regulation Biology of State Education Ministry, College of Life Sciences, Beijing Normal University, Beijing 100875, China; 3 School of Pharmaceutical Sciences, Wuhan University, Wuhan 430071, China; 4 Center for Pharmacogenetics and Department of Pharmaceutical Sciences, University of Pittsburgh, Pittsburgh, PA 15261, USA

**Keywords:** maternal exposure, offspring health, constitutive androstane receptor, TCPOBOP, growth inhibition, lipid absorption

## Abstract

Environmental chemicals, which are known to impact offspring health, have become a public concern. Constitutive activated receptor (CAR) is activated by various environmental chemicals and participates in xenobiotic metabolism. Here, we described the effects of maternal exposure to the CAR-specific ligand 1,4-bis[2-(3,5-dichloropyridyloxy)] benzene (TCPOBOP, TC) on offspring health outcomes. Maternal TC exposure exhibited a stronger inhibition of body weight in 3-week-old and 8-week-old first-generation (F1) offspring female mice compared to controls. Further, maternal TC exposure obtained a strong increase in hepatic drug-metabolizing enzyme expression in 3-week-old female mice that persisted into 8-week-old adulthood. Interestingly, we observed distorted intestinal morphological features in 8-week-old F1 female mice in the TC-exposed group. Moreover, maternal TC exposure triggered a loss of intestinal barrier integrity by reducing the expression of intestinal tight junction proteins. Accordingly, maternal exposure to TC down-regulated serum triglyceride levels as well as decreased the expression of intestinal lipid uptake and transport marker genes. Mechanistically, maternal TC exposure activated the intestinal inflammatory response and disrupted the antioxidant system in the offspring female mice, thereby impeding the intestinal absorption of nutrients and seriously threatening offspring health. Altogether, these findings highlight that the effects of maternal TC exposure on offspring toxicity could not be ignored.

## 1. Introduction

The abnormal trajectory of health-relevant quality of life in industrialized societies is largely attributable to environmental risk factors [1,2,3], as the genetic makeup of the population is unlikely to change dramatically over this time. The industrial revolution in human history has also been accompanied by a drastic increase in air, water, soil pollution and other forms of environmental damage [4,5]. An emerging body of evidence has highlighted that exposure to environmental chemicals would be a crucial factor in the health of organisms [6,7]. Unfortunately, the effects of environmental chemical exposure on the long-lasting health of organisms are currently unknown. Several studies have hypothesized that epigenetics might explain the long-term consequences of gene-environment interactions [8,9,10,11]. Nonetheless, information on the relationship between environmental exposures and adverse health outcomes is scarce.

The potential disruptive effects of environmental chemicals mediated by xenobiotic receptors have received extensive attention from environmental and toxicology scientists. Nuclear receptors, as targets of endogenous or exogenous drugs and chemicals, serve as essential participants in metabolic activity, endocrine signaling and the homeostatic regulation of organisms [12,13]. The nuclear receptor CAR has been found to be activated by structurally diverse exogenous chemicals, including environmental pollutants, drugs, consumer products, and industrial chemicals [14,15,16,17,18,19,20,21,22]. Importantly, CAR, acting as a sensor for endogenous or exogenous compounds, has been considered as a potential target for environmental chemicals to mediate endocrine-disrupting effects. Most in vivo research focusing on CAR-mediated adverse outcome pathways has been performed using a uniquely recognized potent and highly specific mouse CAR ligand, TC [23,24,25,26]. TC is a lipophilic heterocyclic compound originally identified as a pesticide contaminant and also the only known phenobarbital-like agonist of murine-derived CAR [15]. There is still some controversy surrounding the impact of TC-activated CAR on human health. TC-activated CAR could be considered a potential drug target for weight loss [24,27] or the amelioration of colitis [28,29]. Conversely, TC-activated CAR may also induce a variety of adverse events, such as hepatomegaly [30,31,32], nonalcoholic fatty liver disease [33], hepatitis [34], liver cancer [33,35], psoriasis [36] and abnormal milk lipid metabolism [37]. The challenge of explaining the complex relationship between TC exposure and disease across the lifespan remains unclear. Not surprisingly, this lipophilic and extremely long half-life chemical serves as a metabolic disruptor due to its persistence in the organism. It might be transmitted to the next generation and induce poor phenotypes, even if treatment is terminated long before conception [38,39,40,41]. Associated phenotypes might remain undiscovered, as developmental toxicology is now mostly concerned with teratogenic effects. Adverse health outcomes appear in F1 offspring as adults or later in life, possibly through the accumulation and transfer of lipophilic ligands as well as ligand–receptor pair interactions [38]. Accordingly, assessing the impact of TC on the health of the next generation is of great value.

Emerging evidence has revealed that CAR target genes have been found to mediate epigenetic imprinting following CAR-specific activation [42,43,44]. This provides a further implication that CAR-mediated toxicological effects against multiple chemical environmental risks may be transmitted to the next generation. The transient activation of CAR through neonatal exposure to TC led to a permanent increase in drug resistance in the mouse liver. It provides an appropriate example of the association of CAR activation during development with adult health problems [42]. In addition, neonatal exposure to the CAR activator TC and the pregnane X receptor (PXR) activator pregnenolone 16alpha-carbonitrile (PCN) would result in a reduction of the peroxisome proliferator-activated receptor alpha (PPARα) signaling pathway in the adult liver, indicating that neonatal exposure to several xenobiotics might induce certain idiosyncratic lipid metabolism disorders in adulthood [43]. These important findings suggest adverse health effects at the gene expression level associated with TC exposure. Given the bioaccumulation and biomagnification characteristics of xenobiotics, long-lasting exposure and pathological damage to tissue structures might be responsible for the abnormal expression levels of these genes. However, studies on the histomorphological endpoints associated with the effects of TC exposure remain limited. Indeed, the liver and small intestine are highly sensitive tissues when organisms are invaded by harmful xenobiotics. These tissues express a range of xenobiotic processing genes, such as xenobiotic receptors and associated transcription factors, together with phase I and II metabolic enzymes and transporters [45]. From a toxicological viewpoint, the liver and small intestine are interesting tissues for exploring exogenous environmental exposures [46,47]. The effects of TC exposure on the liver have been increasingly recognized, for instance, hepatomegaly [30,32]. Notably, the small intestine is a major site of digestion, nutrient absorption and environmental exposure because of its extensive surface area and physiological properties [48,49]. The intestine is also an essential barrier that separates organisms from the environment contaminated with xenobiotics [50]. Therefore, an intact intestinal structure and function are beneficial for the health of the organism [51]. To date, there is still a lack of knowledge on the changes in offspring intestinal health after maternal exposure to TC. Accordingly, we need more available information to explore the complexity of the relationship between offspring growth and development and intestinal health under the harsh conditions of early-life TC exposure.

As described by the Developmental Origins of Health and Disease (DOHaD) theory [52], extremely slight environmental changes during vital developmental windows could permanently alter the tissue structure, physiological function, and metabolism of offspring. There is growing concern about the effects of prenatal, in utero, or lactational maternal environmental chemical exposures on the next generation. However, the effects of maternal TC exposure on the offspring’s health remain uncertain. Accordingly, in the present study, we mainly assessed changes in body weight, hepatic drug-metabolizing enzymes, and intestinal tract in the F1 generation of maternal exposure to TC. We hope that the results presented below will increase the understanding of the effects of maternal exposure to TC during pregnancy and lactation on the development of the offspring. More importantly, this work would provide new insights into the association between the toxic effects of CAR-targeted environmental chemicals and offspring health.

## 2. Results

### 2.1. Maternal TC Exposure Lowers Body Weight and Reproductive Organ Weight in F1 Offspring Females

**Figure 1:**
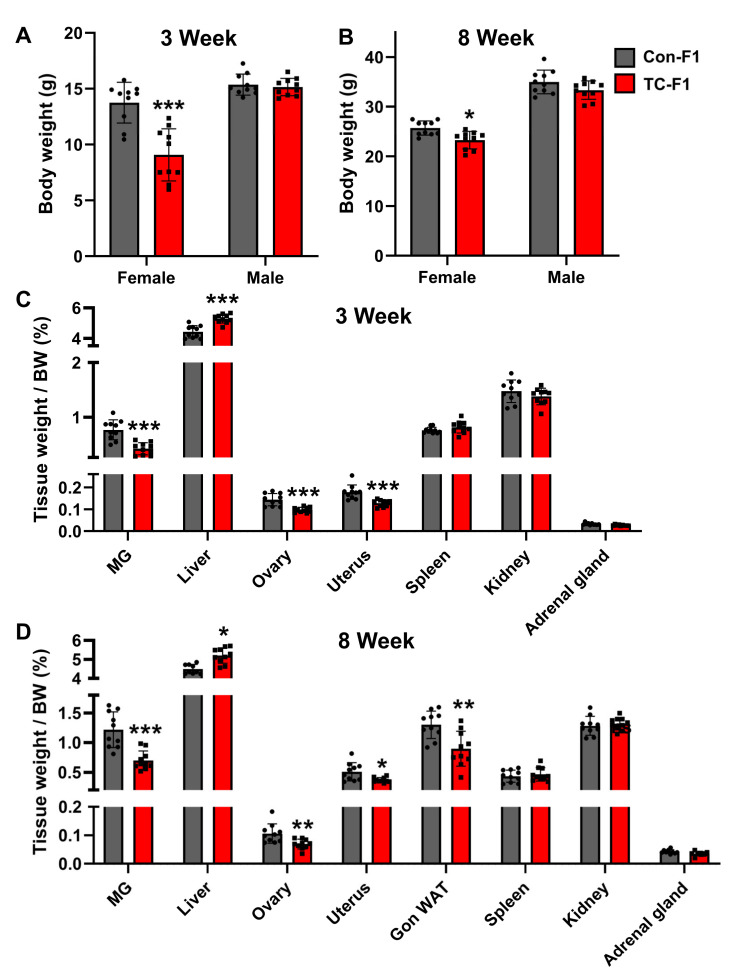
Early-life TC exposure lowers body weight and reproductive organ weight of F1 female offspring. (**A**,**B**) Body weights of 3-week-old (**A**) and 8-week-old (**B**) F1 male and female offspring. (**C**,**D**) The ratio of tissue weight to body weight of 3-week-old (**C**) and 8-week-old (**D**) female F1 offspring. Con-F1: F1 generation of the control group. TC-F1: F1 generation of TC exposure group. *n* = 10. Data are expressed as mean ± SEM. * *p* < 0.05, ** *p* < 0.01, *** *p* < 0.001 vs. Con-F1.

There is considerable interest in investigating the effects of maternal environmental exposures on offspring health. To evaluate whether TC exposure affects offspring growth, we first focused on the body weight of the F1 generation. The results showed that there was no significant difference in the body weight of the 3-week-old and 8-week-old male offspring in the TC-F1 group (Figure 1A,B). As for females, a marked decrease was found at 3 weeks and 8 weeks compared with that of the control group (Figure 1A,B). Analyzing the body weight of 8-week-old F1 female mice, we hypothesized that environmental pollutant exposures in early life may pose a potential long-term threat to offspring health. Combined with previous studies [38,53,54], the effect of TC exposure on female offspring may be more pronounced than those of male offspring. Therefore, our subsequent study focused on the female F1 generation. Interestingly, the relative weights of the mammary gland (MG), ovaries, uterus and gonadal white adipose tissue (Gon-WAT) were significantly reduced in both the 3-week-old and 8-week-old offspring of the TC-F1 group compared with those of the control group (Figure 1C,D). As expected, exposure to TC in early life resulted in a considerable increase in liver weight ratio. Conversely, no significant differences were observed in the spleen, kidney and adrenal glands (Figure 1C,D). Taken together, our findings suggest that TC exposure may lead to adverse health outcomes in female offspring.

### 2.2. Maternal TC Exposure Increases the Expression of Hepatic Drug-Metabolizing Enzymes in Female Offspring Mice

**Figure 2:**
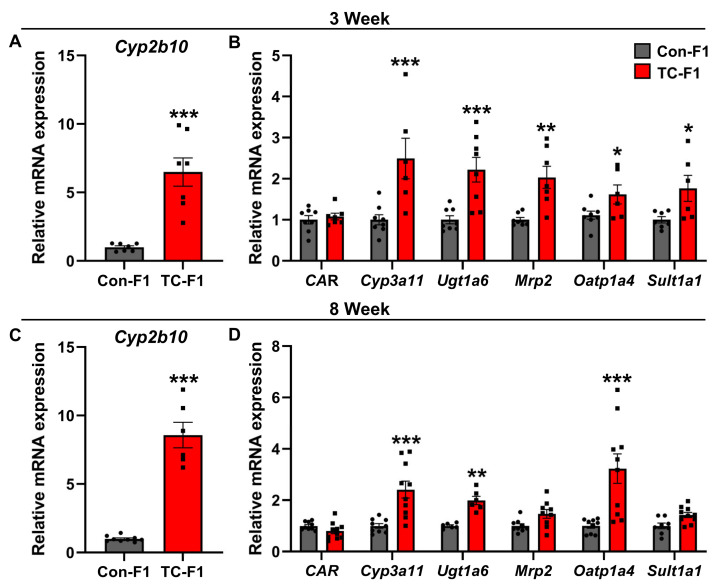
Maternal TC exposure results in abnormal expression of hepatic nuclear receptor and drug-metabolizing enzymes in F1 female mice. (**A**) mRNA expression of hepatic *Cyp2b10* in 3-week-old F1 female mice. (**B**) mRNA expression of hepatic *CAR*, *Cyp3a11*, *Ugt1a6*, *Mrp2*, *Oatp1a4* and *Sult1a1* in 3-week-old F1 female mice. (**C**) mRNA expression of hepatic *Cyp2b10* in 8-week-old F1 female mice. (**D**) mRNA expression of hepatic *CAR*, *Cyp3a11*, *Ugt1a6*, *Mrp2*, *Oatp1a4* and *Sult1a1* in 8-week-old F1 female mice. *n* = 6-10. Data are presented as mean ± SEM. * *p* < 0.05; ** *p* < 0.01; *** *p* < 0.001 vs. Con-F1.

Accumulating evidence illustrates that the activation of CAR by environmental pollutants induces the hepatic expression of genes involved in drug metabolism, leading to epigenetic imprinting that permanently influences health-relevant phenotypes in later life. This prompted us to examine whether CAR-mediated hepatic induction would be transmitted to the F1 generation. In agreement with previous research [38,42,43], we found that the CAR target gene *Cyp2b10* was markedly increased in the livers of the 3-week-old offspring of TC-F1 females (Figure 2A). Additionally, upregulated mRNA expression of hepatic *Cyp3a11*, *Ugt1a6*, *Mrp2*, *Oatp1a4* and *Sult1a1* was seen in the female offspring of the TC-F1 group at 3 weeks after birth compared with the control group (Figure 2B). Consistently, we also found a robust *Cyp2b10* induction in the livers of 8-week-old F1 adult offspring of maternal TC-exposed females (Figure 2C). Similarly, elevated gene expression of hepatic *Cyp3a11*, *Ugt1a6*, and *Oatp1a4* in the TC-F1 group was also found in the 8-week-old female offspring (Figure 2D). In contrast, no statistical differences in the mRNA expression of hepatic *CAR*, *Mrp2* and *Sult1a1* were observed between the TC-F1 group and the Con-F1 group in female offspring (Figure 2D). These findings indicate that the activation of CAR through TC exposure in early life is sufficient to alter the expression of drug metabolism in adolescent age and may exert health-relevant effects on F1 offspring.

### 2.3. Maternal TC Exposure Leads to Nutrient Malabsorption in Offspring

**Figure 3:**
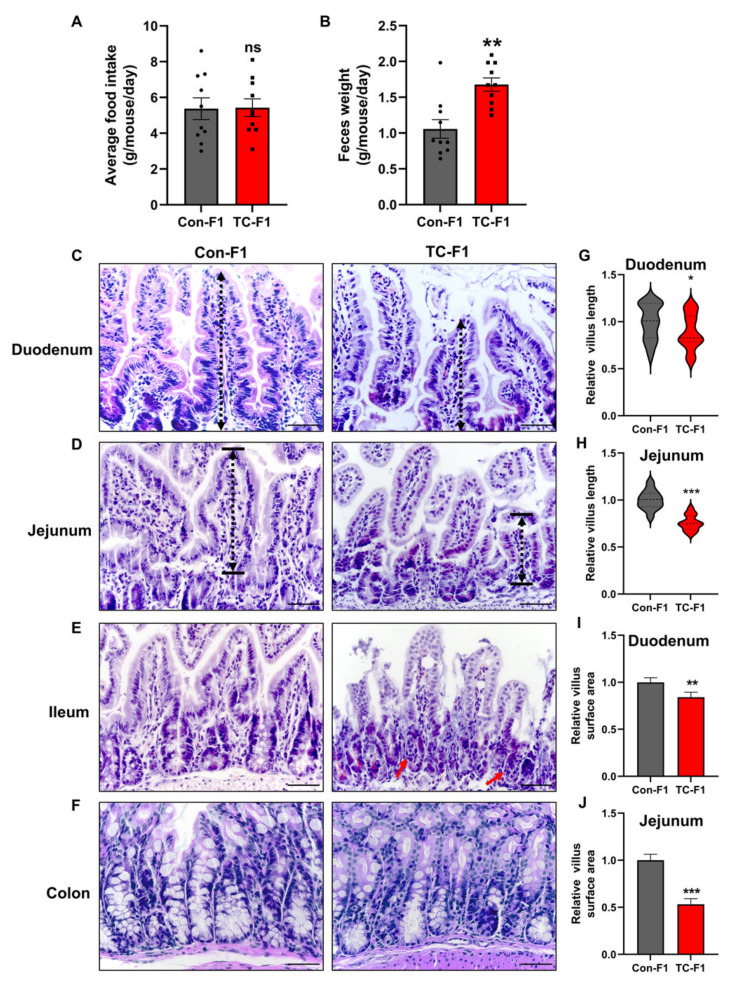
Maternal TC exposure affects small intestinal morphology in 8-week-old F1 female mice. (**A**) Average daily food intake of 8-week-old F1 female mice. (**B**) Average daily fecal production of 8-week-old F1 female mice. *n* = 10. (**C**–**F**) Representative HE images of the duodenum (**C**), jejunum (**D**), ileum (**E**), and colon (**F**) of 8-week-old offspring female mice. Black dashed lengths represent intestinal villi lengths and red arrows represent inflammatory cell aggregates. Scale bar = 50 μm. (**G**,**H**) Relative duodenal (**G**) and jejunal villus length (**H**). (**I**,**J**) Relative villi surface area of the duodenum (**I**) and jejunum (**J**). Data are presented as mean ± SEM. * *p* < 0.05; ** *p* < 0.01; *** *p* < 0.001 vs. Con-F1.

To explore possible reasons for weight loss in adolescent offspring, we next focused on food intake in mice. As depicted in Figure 3A, the 8-week-old female offspring of the TC-F1 group revealed a normal daily food intake. However, food intake is not equivalent to the efficiency of nutrient assimilation. From the perspective of nutritional balance, weight loss may be due to decreased nutrient intake or increased energy output. Accordingly, there may be nutrient malabsorption in the TC-F1 group, which may be resulting in weight loss. To test this hypothesis, we next monitored daily feces production. As expected, the TC-F1 group exhibited increased daily fecal production compared to the control group (Figure 3B). These results suggest that intestinal nutrient malabsorption could contribute to considerable differences in body weight in the long term. It is well known that the intestinal morphology structure reflects nutrient absorption to some degree. As villi length is usually used to evaluate intestinal growth [55], we investigated morphological expressions to assess intestinal growth in both groups of offspring. H&E staining revealed that the duodenal villus length and its surface area were remarkably restricted in 8-week-old female offspring of the TC-F1 group compared to those in the control group (Figure 3C,G,H). This indicates that maternal TC exposure may affect the effective absorption of nutrients in the duodenum of the offspring. Similar findings were observed in the jejunum (Figure 3D). Meanwhile, the jejunal villi in the TC-F1 group exhibited unusual changes, including partial loss of shape and shedding (Figure 3D,I,J), suggesting that early-life TC exposure may result in jejunal epithelial injury. Moreover, the ileum showed a slight inflammatory infiltration in 8-week-old female offspring of maternal TC exposure (Figure 3E). Furthermore, no obvious signs of pathological damage were observed in the colon of F1-generation female mice in both groups (Figure 3F). Collectively, these results suggest that the unusual changes in small intestinal morphology associated with nutrient malabsorption might account for the lower body weight in TC-exposed offspring females.

### 2.4. Maternal TC Exposure Compromises the Intestinal Mucosal Barrier of Offspring

**Figure 4:**
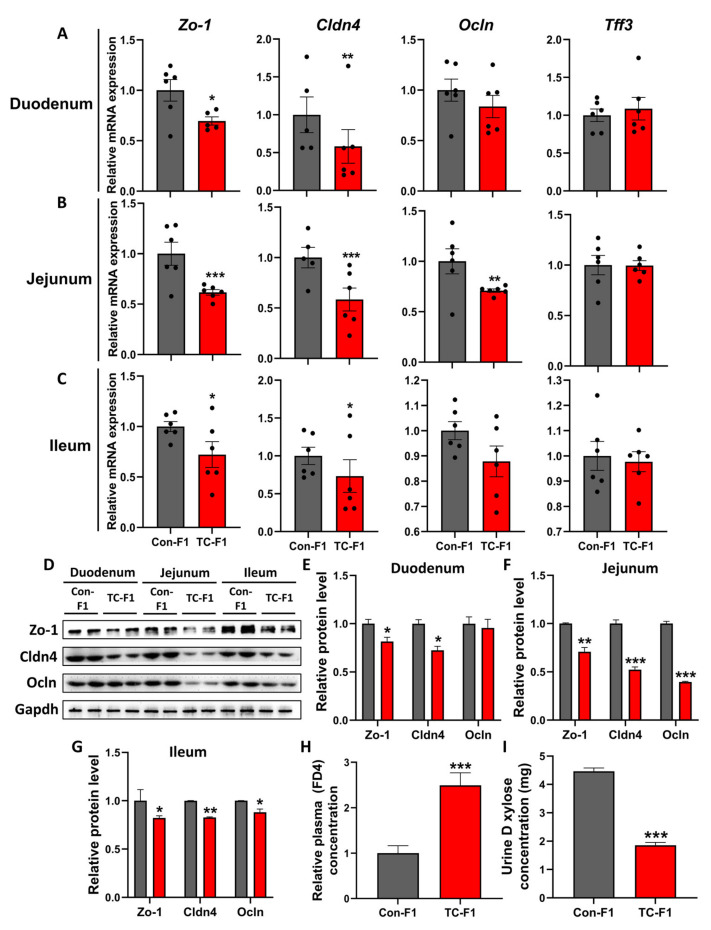
Early-life TC exposure compromises intestinal mucosal barrier integrity in female offspring. (**A**–**C**) *Zo-1*, *Cldn4*, *Ocln* and *Tff3* mRNA expression in the duodenum (**A**), jejunum (**B**) and ileum (**C**) of 8-week-old offspring female mice, *n* = 5-6. (**D**) Zo-1, Cldn4 and Ocln protein levels in duodenum, jejunum and ileum of 8-week-old offspring female mice. (**E**–**G**) Quantitative statistics of Zo-1, Cldn4 and Ocln protein levels in the duodenum (**E**), jejunum (**F**) and ileum (**G**). (**H**) Relative concentration of plasma FITC (FD4), *n* = 10. (**I**) Urinary D-xylose concentration, *n* = 10. Data are expressed as mean ± SEM. * *p* < 0.05, ** *p* < 0.01, *** *p* < 0.001 vs. Con-F1.

The intestinal epithelial barrier plays a crucial role in the absorption and utilization of nutrients, as well as in defense against environmental stimuli [56,57]. Attempting to understand the mechanisms responsible for nutrient malabsorption in TC-exposed offspring, we assessed the expression of crucial genes associated with intestinal barrier functions of the 8-week-old female offspring. The results showed that mRNA levels of *Zo-1* and *Cldn4* were significantly downregulated in small intestine (duodenum, jejunum and ileum) samples of the TC-F1 group (Figure 4A–C). Notably, the mRNA levels of *Ocln* were significantly decreased in the jejunum of offspring after maternal TC exposure (Figure 4B). Differently, mRNA levels of other genes (*Ocln* and *Tff3*) were not statistically different in the duodenum and ileum (Figure 4A,C). Similarly, no difference in mRNA expression of *Tff3* was found in the jejunum (Figure 4B). Interestingly, Zo-1, Cldn4 and Ocln protein levels in small intestine samples also showed consistent findings (Figure 4D–G). Furthermore, macromolecular permeability, as demonstrated by increased mucosal-to-serosa transport of 4 KDa FITC-labeled dextran, was elevated in the TC-F1 group (Figure 4H). Additionally, the concentration of D-xylose in urine was markedly decreased in the TC-F1 group (Figure 4I), indicating that the barrier of intestinal mucosa was significantly impaired. Together, these findings indicate that TC exposure in early life could affect the intestinal barrier function of offspring, which may interfere with nutrient absorption in the small intestine, resulting in poor health outcomes in the offspring.

### 2.5. Maternal TC Exposure Impairs Lipid Absorption within the Small Intestine of Offspring

**Figure 5:**
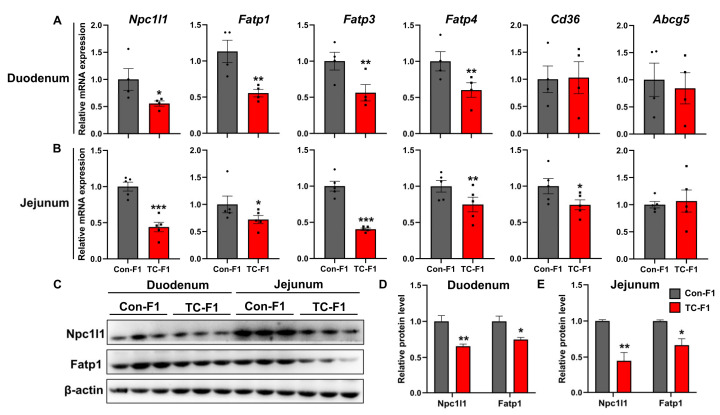
TC exposure in early life impairs the expression of intestinal lipid-related transporter genes in female offspring. (**A**,**B**) mRNA expression of *Npc1l1*, *Fatp1*, *Fatp3*, *Fatp4*, *Cd36* and *Abcg5* in the duodenum (**A**) and jejunum (**B**) of 8-week-old offspring female mice, *n* = 4–5. (**C**) Npc1l1 and Fatp1 protein levels in duodenum and jejunum of 8-week-old offspring female mice. (**D**,**E**) Quantitative statistics of Npc1l1 and Fatp1 protein levels in duodenum (**D**) and jejunum (**E**). *n* = 3. Data are presented as mean ± SEM. * *p* < 0.05, ** *p* < 0.01, *** *p* < 0.001 vs. Con-F1.

As we showed that maternal TC exposure induced intestinal barrier impairment related to nutrient malabsorption, we further found that the TG levels of 8-week-old female offspring in serum had an obvious down-regulation in the TC-F1 group (Table 1). Based on these findings, we hypothesized that weight loss in TC-exposed female offspring is associated with active impairment in lipid absorption. Accordingly, we sought to further investigate whether TC-exposed offspring exhibit impaired lipid absorption and to determine the mechanisms involved. Given that the distal part of the duodenum and the upper part of the jejunum serve as the crucial site of lipid absorption, we focused on the expression of lipid absorption marker genes in these two intestinal segments. As seen in Figure 5A, decreased mRNA expression of *Npc1l1*, *Fatp1*, *Fatp3* and *Fatp4* was observed in the duodenum of the TC-F1 group, whereas the expression of *Cd36* and *Abcg5* was not statistically different. Notably, except for gene *Abcg5*, the expression of which was not influenced by TC exposure, the expressions of the other five genes (*Npc1l1*, *Fatp1*, *Fatp3*, *Fatp4* and *Cd36*) were significantly decreased in the jejunum of the TC-F1 group (Figure 5B). Further confirming this observation, we next examined the protein expression levels of Npc1l1 (a protein that regulates cholesterol absorption) and Fatp1 (a fatty acid transporter protein). As expected, significantly lower protein levels of Npc1l1 and Fatp1 were observed in the duodenum and jejunum in the TC-F1 groups (Figure 5C–E). Taken together, our data indicate that TC exposure in early life could result in a decreased ability of offspring to absorb lipids in the intestine, which would be associated with the restricted expression of genes involved in regulating intestinal lipid transport.

### 2.6. Maternal TC Exposure Triggers Intestinal Inflammation and Oxidative Stress in Offspring

**Figure 6:**
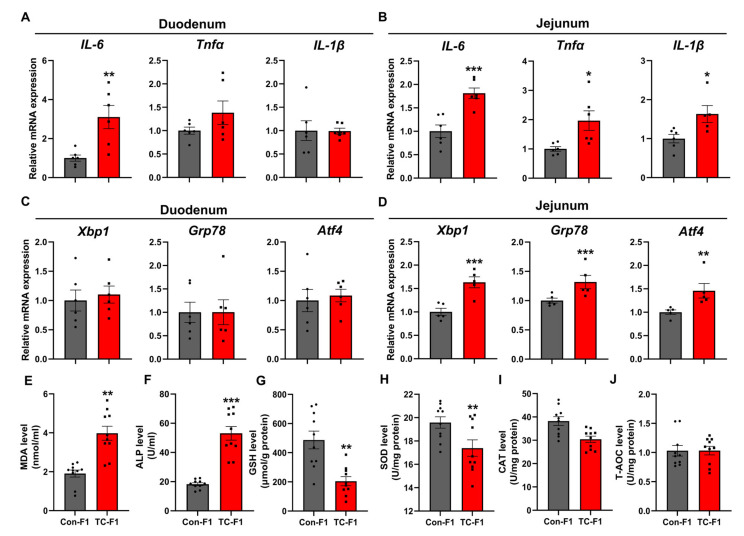
TC exposure in early life results in slight inflammation and oxidative stress in the jejunum of female offspring. (**A**,**B**) mRNA expression of inflammatory markers *IL-6*, *Tnfα* and *IL-1β* in the duodenum (**A**) and jejunum (**B**) of 8-week-old offspring female mice, *n* = 5-6. (**C**,**D**) mRNA expression of oxidative stress marker genes *Xbp1*, *Grp78* and *Atf4* in the duodenum (**C**) and jejunum (**D**) of 8-week-old offspring female mice, n = 5-6. (**E**–**J**) Levels of MDA (**E**), ALP (**F**), GSH (**G**), SOD (**H**), CAT (**I**) and T-AOC (**J**) markers associated with oxidative stress, in the jejunum of 8-week-old female offspring mice, *n* = 10. Data are expressed as mean ± SEM. * *p* < 0.05, ** *p* < 0.01, *** *p* < 0.001 vs. Con-F1.

We next asked about the mechanisms behind decreased lipid absorption within the small intestine in the TC-F1 group. Obtained results have shown that structural damage to the intestinal mucosa and abnormal lipid absorption often result from the dysregulation of the expression of relevant inflammatory cytokines or the promotion of oxidative stress in the intestine [58,59,60,61,62,63]. Therefore, we characterized the levels of inflammation and oxidative stress in the intestine of the offspring of both groups. As shown in Figure 3C, no significant inflammatory cells were found in the duodenum and jejunum in H&E staining. However, we observed a marked increase in the mRNA levels of *IL-6* in the duodenum and jejunum of the TC-F1 group (Figure 6A,B). Additionally, the mRNA levels of *Tnfα* and *IL-1β* in the jejunum were also significantly up-regulated in the TC-F1 group (Figure 6B). In contrast, no statistical difference was found in *Tnfα* and *IL-1β* mRNA expression in the duodenum in the TC-F1 group (Figure 6A). These results suggest that TC exposure in early life could lead to slight inflammation in the offspring intestine, especially in the jejunum. Numerous studies have revealed that intestinal inflammatory injury is tightly linked to oxidative stress [64,65,66,67,68,69]. In our previous results, we found a significant increase in serum ALT levels in the TC-F1 group (Table 1). In addition, the serum levels of AST showed an increasing trend and did not have a statistical difference in the TC-F1 group (Table 1). It is well known that elevated ALT and AST activity is not only a sign of abnormal liver function, but also a key marker of stress response [70,71,72]. Accordingly, we next focused on oxidative stress-related markers. Our results showed that the mRNA expression of *Xbp1*, *Grp78* and *Atf4*, the target genes associated with oxidative stress, were not significantly different in the duodenum of the TC-F1 group compared with the control group (Figure 6C). However, these three target genes were markedly upregulated in the jejunum (Figure 6D). This result is consistent with the expression of inflammatory factors, implying that TC exposure led to more pronounced oxidative stress induced in the jejunum of F1-generation female mice in response to environmental stress. We further examined biochemical markers associated with oxidative stress in the jejunum. We found that the levels of MDA (Figure 6E) and ALP (Figure 6F) were considerably upregulated in the jejunum of the TC-F1 group compared with the control group. However, the levels of GSH (Figure 6G) and SOD (Figure 6H) were remarkably down-regulated, and there were no significant differences in CAT (Figure 6I) and T-AOC (Figure 6J). These results suggest that TC exposure in early life may affect the levels of MDA, ALP, GSH and SOD in the offspring jejunum, leading to oxidative stress in the jejunum.

## 3. Discussion

**Figure 7:**
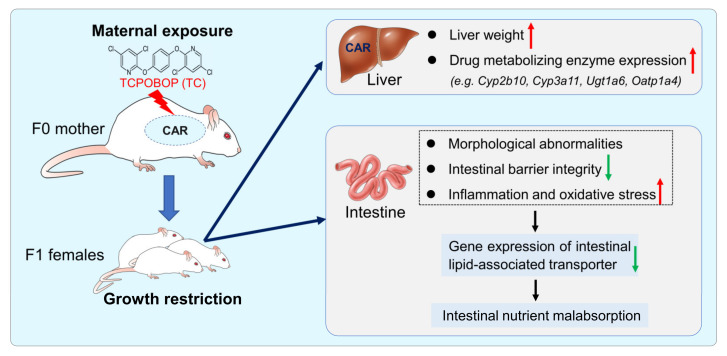
Schematic illustration of maternal TC exposure and offspring health. Maternal exposure to TC resulted in decreased body weight and upregulated expression of drug-metabolizing enzymes in the liver of 3-week-old and 8-week-old F1 female mice. In addition, TC exposure during early life resulted in abnormal small intestinal structure and impaired mucosal barrier in 8-week-old F1 female mice, stimulating slight inflammation and oxidative stress in the intestine. Accordingly, the ability of the intestine to absorb lipids was diminished in the 8-week-old female mice in the TC-exposed group, which led to growth restriction in the offspring. Red arrows indicate up-regulation, and green arrows indicate down-regulation.

According to the developmental origins of health and disease theory, environmental factors such as drugs, nutrients, and infections are particularly sensitive during critical periods of individual development, including intrauterine development, neonatal and childhood. This leads to the altered expression of marker genes associated with physiological functions, increasing susceptibility to chronic diseases in adulthood and affecting long-term health. In this study, we found that TC exposure in early life could affect the mucosal barrier in the small intestine of offspring, resulting in slight inflammation and oxidative stress in the intestine, further weakening the ability of the intestine to absorb lipids. Malabsorption of nutrients leads to restricted growth and development of the offspring (Figure 7).

It is well known that exposure to adverse factors in early life has potential effects on health in adulthood. Previous studies have shown that transient neonatal exposure to TC induces CAR activation while leading to the reprogramming of epigenetic information and a permanent increase in hepatic drug resistance in mice [42], suggesting a dramatic impact of CAR activation on adult health. However, large gaps remain in revealing the offspring’s developmental toxicity and the mechanisms triggered by environmental exposures. Our previous work showed that TC exposure could affect mammary gland development in adolescent mice [73], and we unexpectedly found an effect of TC exposure on offspring body weight. Accordingly, whether TC exposure affects the health of offspring was our next concern. As expected, our results revealed that the body weight of female F1-generation mice in the TC-exposed group was considerably lower than that of the control group at both 3 and 8 weeks old. In contrast, there was no significant difference in the body weight of male F1 generation mice. This observation is consistent with previous studies [53,54]. Females were more sensitive to CAR agonists, such as TC, compared to males [74]. The possible reason is that androgens could inhibit the activity of CAR, while estrogen could act as an agonist of CAR, which would increase the activity of CAR [75,76]. Together, the effect of TC on female offspring may be more pronounced than that on male offspring. In addition, the growth and development of 3-week-old F1 offspring are known to be closely related to maternal milk nutrition. Our previous work indicated that TC preferred lactation performance to interfere with offspring growth [37]. In the present study, we found that 3-week-old female offspring in the TC-exposed group showed a significant reduction in body weight. Combined with these findings, the contribution of maternal milk composition or pup suckling behavior to F1 offspring of different sexes remains to be explored. However, the pups were not treated with TC directly after weaning. Under the same experimental conditions, the body weight of 8-week-old F1 females in the TC-exposed group was markedly lower. These observations indicate that environmental exposure in early life may pose a potential long-term threat to the health of the offspring, and the mechanisms involved need to be further elaborated.

Similarly, we focused on the expression of drug-metabolizing enzyme-related genes (*Cyp2b10*, *CAR*, *Cyp3a11*, *Ugt1a6*, *Mrp2*, *Oatp1a4* and *Sult1a1*) in the livers of offspring female mice. As expected, TC exposure somehow persistently altered the expression of genes associated with drug-metabolizing enzymes in mice from birth to adulthood. This also explores whether maternal exposure to TC affects the expression of genes involved in drug-metabolizing enzymes in the livers of offspring females at different ages from the perspective of the developmental origins of health and disease theory, with a view to filling the cognitive gap regarding the long-term effects of maternal environmental exposures on drug metabolism and the health of offspring.

The intestine is the major site of nutrient absorption and transport. Detrimental impacts on intestinal function in response to environmental exposure are becoming increasingly evident. Changes in intestinal function have been found to have long-term effects on adolescent health, so studying intestinal physiology following TC exposure is timely. The integrity of the intestinal mucosal barrier is known to be critical for nutrient absorption [77]. The abnormalities in intestinal morphology induced by exposure to TC in early life observed in our work may have implications for the intestinal barrier. Previous studies have shown that the impaired function of tight junction protein compositions is highly linked to elevated paracellular permeability and the development of multiple intestinal diseases [78,79,80]. For example, *Zo-1* insufficiency may contribute to intestinal permeability and dysfunction as it recruits other tight junction proteins [81,82]. In our work, the dysregulation of intestinal tight junction marker genes, such as *Zo-1*, *Cldn4*, and *Ocln*, further indicated that an imbalance of the tight junctions exacerbated nutrient absorption dysfunction. Combined with the finding of decreased serum TG levels in the TC-exposed group, we speculated that the weight loss may be related to dysregulated intestinal lipid absorption. Given that the duodenum and jejunum are the main sites of lipid absorption, we focused on the expression of lipid absorption genes in these intestinal segments. Fortunately, we found that the key genes for lipid absorption (*Npc1l1*, *Fatp1*, *Fatp3*, *Fatp4*) expressed in the duodenum and jejunum of the TC-exposed group were obviously suppressed, implying that the inhibition of fatty acid transport and lipid adsorption might be responsible for the weight loss in the TC-exposed group in early life. Furthermore, alterations in genes associated with inflammatory (*IL-6*, *Tnfα* and *IL-1β*) and antioxidant systems (*Xbp1*, *Grp78* and *Atf4*) further provided insights into intestinal dysfunction induced by TC exposure. Structural and functional impairments may be the result of an imbalanced redox state in the jejunum of the offspring female mice. The up-regulation of MDA and ALP and the down-regulation of GSH and SOD contents, together with histopathological damage illustrated that the oxidative stress induced by TC exposure in early life led to intestinal structural disorders and dysfunction in female offspring. This has further inhibited lipid digestion and absorption, resulting in growth restriction of the offspring.

Our study demonstrated a prominent effect of early-life TC exposure on female offspring body weight, suggesting that intestinal nutrient malabsorption may be involved. Notably, several limitations should be considered. To begin with, while early-life TC exposure was negatively associated with serum TG concentrations in offspring mice, the mechanisms of how TC interferes with lipid metabolism have not been investigated. In the next study, we will look at how early-life TC exposure affects the intestinal lipid absorption of the offspring, with the aim of understanding the reasons for the growth restriction of the offspring. Furthermore, the causes of gender differences are unclear and should be further explored. For example, the integral role of sex hormones in offspring growth should be explored. Moreover, we only examined the effects of successive TC exposure (including pregnancy and lactation) on offspring, but we did not investigate which period is more critical. Lastly, our present work is a relatively descriptive study. Human-derived hepatic CAR is highly homologous to murine CAR at the protein level [83], and its function is nearly similar to that of mouse CAR. However, it has been reported that, as the phenotype that appears in mice following murine-derived CAR activation, human-derived CAR-specific activation does not necessarily elicit the same response in human or transgenic mice [84,85]. Our study focused on the effects of TC activation of murine-derived CAR-specific agonist ligands on offspring growth, and the extrapolation of data to human studies must be undertaken with caution due to species differences. These biological mechanisms and public health implications would be verified by additional studies in the future. Similarly, genetic susceptibility and epigenetic changes remain to be discovered. It will be valuable to consider using CAR knockout or humanized mouse models to explain these questions in future studies.

Taken together, we described how early-life TC exposure might lead to poor health-relevant phenotypes in offspring. These findings link TC exposure to offspring growth and constitute a resource for further research into the effects of environmental chemistry on offspring health.

## 4. Materials and Methods

### 4.1. Animals and Experimental Design

All animal protocols in this study have been approved by the Ethics and Animal Welfare Committee of Beijing Normal University (No. Approval No. CLS-EAW-2015-006, date: 5 May 2015, and CLS-EAW-2020-004, date: 16 March 2020). The CD1 (ICR) male and female mice were purchased from Vital River Laboratory Animal Technology Co. Ltd. (Beijing, China). All experimental mice were allowed to drink water and food freely, and were housed at 22–25 °C, 50–60 % relative humidity, and with a 12 h light–dark lighting cycle.

The parental 8-week-old mice were randomly divided into control (Con) and TC-exposed groups after 1 week of adaptive feeding. In the TC exposure group, female mice were exposed to TC (0.5 mg/kg) by intraperitoneal injection, twice a week [73]. In the Con group, female mice were intraperitoneally injected with saline in the same manner. Female mice were exposed to TC for 2 weeks prior to pregnancy and continued until the end of lactation, but male mice were not treated with TC throughout the experiment, as described previously [37]. Offspring were weaned 21 days postpartum, and five offspring were housed in same-sex groups without access to TC. At the corresponding experimental endpoints, 3 weeks and 8 weeks old, F1 offspring mice were euthanized by CO_2_. One male and one female mouse per litter were selected to avoid maternal effects. Various tissues were also collected, including the mammary gland, uterus, ovary, liver, kidney, intestine, spleen, and adipose. The tissues were divided into two parts, one for gene and protein expression analysis, and stored in liquid nitrogen or −80 °C. The other was used for histological analysis and stored in 4% paraformaldehyde. The experimental procedure was shown in Supplementary Figure S1.

### 4.2. Serum Biochemical Analysis

Blood samples were taken from the orbits. Serum was collected by centrifugation at 3000× g for 15 min at 4 °C. Triglyceride (TG), total cholesterol (TC), alanine transaminase (ALT), aspartate aminotransferase (AST), creatinine (CREA), urea nitrogen (UREA), uric acid (UA), and γ-glutamyltransferase (γ-GT) levels were determined using a Hitachi 7600 automated biochemical analyzer (Diamond Diagnostics Inc., Holliston, MA, USA).

### 4.3. Antioxidant Activity Assay

The activities of microdermabrasion (MDA), alkaline phosphatase (ALP), glutathione (GSH), superoxide dismutase (SOD), catalase (CAT), and total antioxidant capacity (T-AOC) in jejunum tissue were assessed using a commercially available kit (Nanjing Jiancheng Bioengineering Institute, Nanjing, China). These procedures are performed according to the manufacturer’s protocols.

### 4.4. Plasma Fluorescent Measurement

Mice were fasted for 4 h and gavaged with fluorescently labeled FITC-dextran 4 kD at a dose of 20 mL/kg. Blood was taken from the orbit after 3 h and centrifuged at 3000× *g* for 15 min to collect plasma. Plasma was diluted 1:2 (*v*/*v*) with PBS and 100 μL of diluted standards and plasma was transferred to a black 96-well plate. The fluorescence intensity of plasma was measured using a fluorescence reader (excitation: 490 nm, emission: 520 nm).

### 4.5. Urine D-Xylose Content Assay

Mice fasted for 16 h overnight, and urine of each mouse was collected the next morning as blank urine. Subsequently, each mouse was given 10% D-xylose at a dose of 1.5 g/kg. Five hours later, the urine of each mouse was collected as the endpoint urine. Food and water were freely available in this experiment. The urine D-xylose content was determined using a commercially available kit (Nanjing Jiancheng Bioengineering Institute, Nanjing, China). These procedures were performed in accordance with the manufacturer’s instructions.

### 4.6. Histological Analysis

For histological and morphometric analysis, tissue samples were fixed in 4% paraformaldehyde and embedded in paraffin, sectioned at 5 µm and stained with hematoxylin and eosin (H&E). H&E staining was carried out according to the method we used previously [86]. Briefly, hematoxylin solution was stained for 3 min and eosin for 1 min.

### 4.7. Gene Expression Analysis

Total RNA was isolated using the RNAprep Pure tissue kit (Tiangen, Beijing, China) and reverse transcribed to cDNA using the FastKing gDNA Dispelling RT SuperMix (Tiangen, Beijing, China). For RNA quantification, we used SYBR Green qPCR SuperMix (Transgen Biotech, Beijing, China) and ran the reaction on an ABI Q6 instrument (Thermo Fisher Scientific, Waltham, MA, USA) according to the manufacturer’s protocols. The mRNA levels were normalized to 18s expression by the comparative CT method. Information on qPCR primers was shown in Supplementary Table S1.

### 4.8. Western Blot Analysis

Tissue protein samples were homogenized in RIPA lysis buffer (Applygen Technologies Inc., Beijing, China) containing 1 mmol/L PMSF (Sigma Aldrich, St. Louis, MO, USA). Equal amounts of proteins were separated on 10% running gels using sodium dodecyl sulfate-polyacrylamide gel electrophoresis (SDS-PAGE) and then transferred to polyvinylidene difluoride membranes. The membranes were blocked with 5% low-fat dry milk in Tris-buffered saline (TBST) supplemented with 0.1% Tween-20 for 1 h at room temperature. Thereafter, membranes were incubated with primary antibodies overnight at 4 °C. Secondary antibodies were diluted 1:5000 for 1 h at room temperature. Information about the antibodies is listed in Supplementary Table S2.

### 4.9. Statistical Analysis

Statistical analysis was assessed using Prism 9 software. Differences between groups were made with a two-tailed Student’s *t*-test. All error bars are expressed as standard error (SEM). Experimental data are reported as the mean values ± SEM. A value of *p* < 0.05 was considered significant.

## 5. Conclusions

The health effects of offspring associated with environmental chemicals are one of the major concerns worldwide. CAR, a key exogenous sensor, is activated by a variety of environmental chemicals. However, the effect of exposure to CAR-specific ligand TC on offspring health outcomes remains inconclusive so far. The results demonstrated that maternal exposure to TC resulted in decreased body weight and upregulated expression of drug-metabolizing enzymes in the livers of 3-week-old and 8-week-old F1 female mice. More importantly, TC exposure during early life resulted in damage to the small intestinal mucosal barrier in 8-week-old F1 female mice, stimulating inflammation and oxidative stress in the intestine. Accordingly, the ability of the intestine to absorb lipids was diminished in the 8-week-old female mice in the TC-exposed group, which ultimately led to poor health outcomes in the offspring. These results suggest that early life, an important period of neonatal growth and development, is vulnerable to disturbances in environmental exposure and may have profound effects on the health of the offspring.

## Figures and Tables

**Table 1:** Serum biochemical parameters.

Parameter	Con-F1	TC-F1
**TG (mmol/L)**	1.59 ± 0.10	0.84 ± 0.06 ***
**TC (mmol/L)**	2.68 ± 0.14	2.94 ± 0.33
**ALT (U/L)**	48.03 ± 4.26	112.95 ± 5.03 ***
**AST (U/L)**	184.23 ± 10.78	213.09 ± 14.92
**CREA (** **μmol/L)**	37.00 ± 0.64	37.53 ± 0.96
**UREA (mmol/L)**	13.75 ± 0.37	13.18 ± 0.25
**UA (** **μmol/L)**	91.91 ± 16.04	103.09 ± 8.66
**γ-GT (U/L)**	70.50 ± 3.67	64.27 ± 4.01

Triglyceride (TG), total cholesterol (TC), alanine transaminase (ALT), aspartate aminotransferase (AST), creatinine (CREA), urea nitrogen (UREA), uric acid (UA), and γ-glutamyl transpeptidase (γ-GT). Data are expressed as mean ± SEM. *n* = 8–10. *** *p* < 0.001 vs. Con-F1.

## Data Availability

Data are contained within the article and Supplementary Material.

## Supplementary Materials

The following supporting information can be downloaded at: https://www.mdpi.com/article/10.3390/ijms24032602/s1.
