# Supplementary figures and images for: Constitutive Androstane Receptor Agonist, TCPOBOP: Maternal Exposure Impairs the Growth and Development of Female Offspring in Mice
Source: Int J Mol Sci. 2023 Jan 30;24(3):2602. doi: 10.3390/ijms24032602 (PMC9917268; doi:10.3390/ijms24032602)

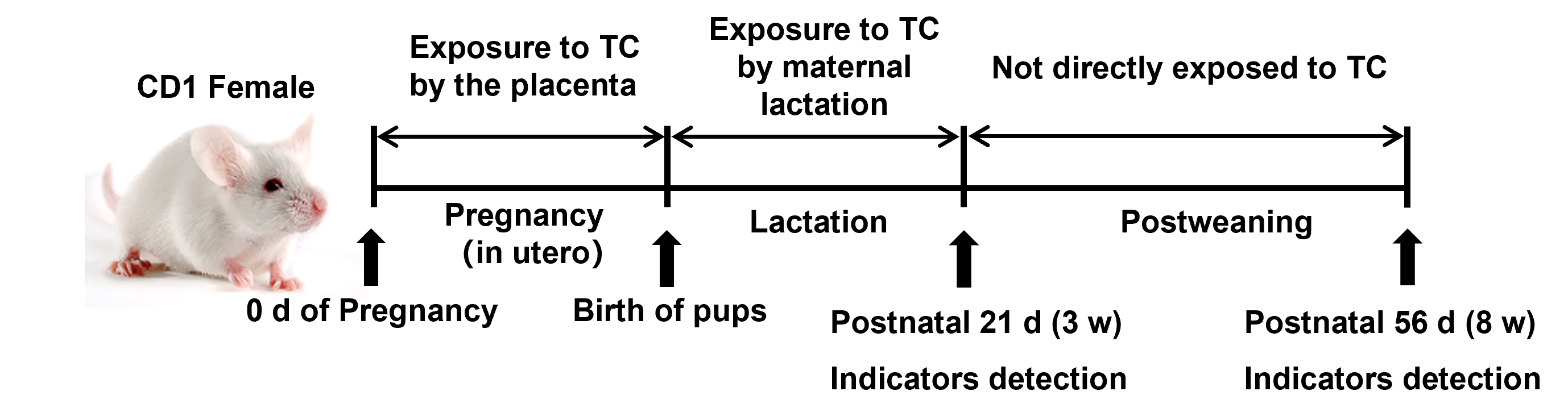

Supplement: Supplementary file 1 [file ijms-24-02602-s001.zip › Figure S1-Conceptual graph of the experimental procedure.tif]
